# Primary Obstructive Megaureter with Giant Ureteral Stone: A Case Report

**DOI:** 10.1155/2013/198592

**Published:** 2013-02-27

**Authors:** Abdullah Demirtaş, Nurettin Şahin, Emre Can Akınsal, Oguz Ekmekçioğlu, Atila Tatlışen

**Affiliations:** Department of Urology, Erciyes University Medical Faculty, 38039 Kayseri, Turkey

## Abstract

A 19-year-old male patient was admitted with flank pain, which had lasted intermittently for four years. In X-ray, there was a radiopacity with a dimension of 6 × 4 cm on the left pelvic bone. Intravenous pyelography revealed a huge left megaureter with a stone in the lower end and grade V hydronephrosis. A left ureterolithotomy, left nipple ureteroneocystostomy, and psoas hitch operation was performed. A voiding cystourethrogram taken three months after the operation showed no reflux, and in IVP there was reduced dilatation of the collecting system when compared to the ureter before the operation.

## 1. Introduction

Primary obstructed megaureter (POM) is a result of obstruction or an adynamic ureteral segment in the ureterovesical junction [1]. POM occurs more often in males [2]. Although it is generally unilateral, it may be seen bilaterally in 15% to 25% of cases. Besides excretory urography (IVP), diuretic renography and antegrade pyelography are valuable tools for its diagnosis. As treatment, the adynamic ureteral segment is excised and ureteroneocystostomy is performed with different surgical techniques. However, the results may be far from ideal [3, 4]. We present a case of primary obstructed megaureter with a giant ureteral stone.

## 2. Case Report

**Figure 1:**
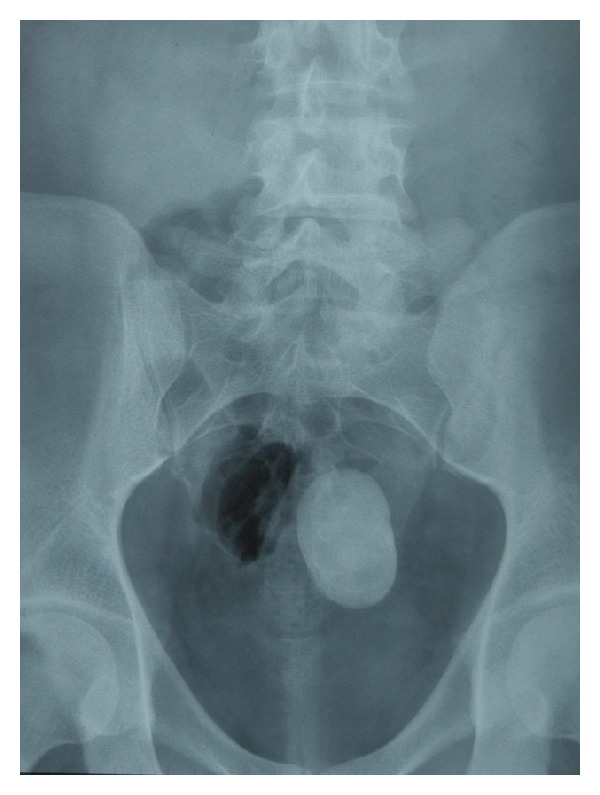
Preoperative direct urinary system radiography.

A 19-year-old male was admitted with the complaint of intermittent flank pain with a history of four years. There was no record of stones in his history. The systemic and urogenital examination was normal. In his direct urinary system radiography there was a radiopacity with a dimension of 6 × 4 cm on the left pelvic bone (Figure 1).

**Figure 2:**
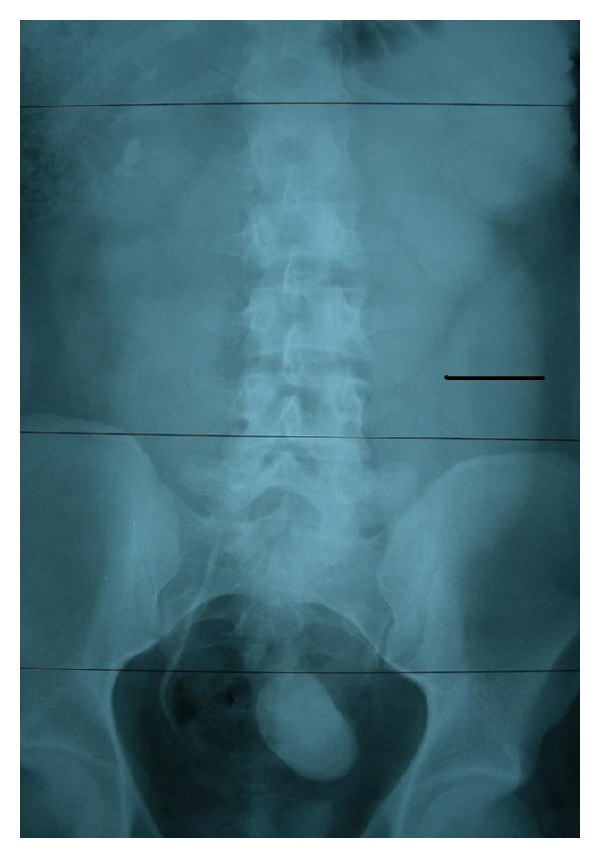
Preoperative intravenous pyelography.

In intravenous pyelography (IVP) advanced hydroureteronephrosis (grade V) was seen and there was no transit of the radiopaque dye through the bladder. In IVP it was determined that the opacity belonged to the lower end of the left ureter (Figure 2).

**Figure 3:**
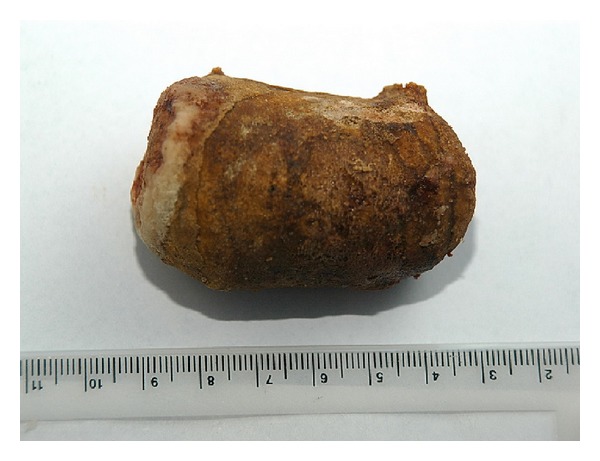
A giant ureter stone.

In abdominal ultrasonography (USG) it was observed that the left calyceal structures and pelvis were extremely dilated and the left ureter had dilated from the proximal end through the distal end. Left kidney function was 39.6% in 99 m Tc-dimercaptosuccinic acid (DMSA). The renal function test and electrolyte values were normal in serum analysis. A direct nipple ureteroneocystostomy and ureterolithotomy operation was performed with a left Gibson incision as reported previously [5]. During surgery it was seen that the left ureter wall had become quite thickened and the left ureter had dilated. A giant ureter stone with dimensions of approximately 6 × 5 × 4 cm and weighing 61 gr. was extracted from the left ureter (Figure 3).

**Figure 4:**
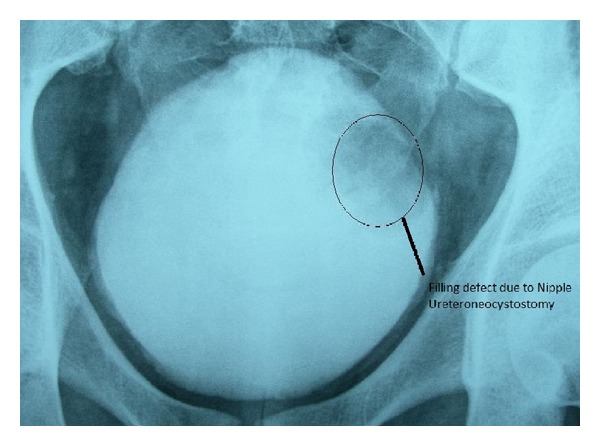
Postoperative voiding cystography.

At the patient's follow-up examination in the postoperative third month there was no proliferation in his urine culture. A postoperative voiding cystourethrogram showed no reflux and the appearance of a filling defect due to the left nipple (Figure 4).

**Figure 5:**
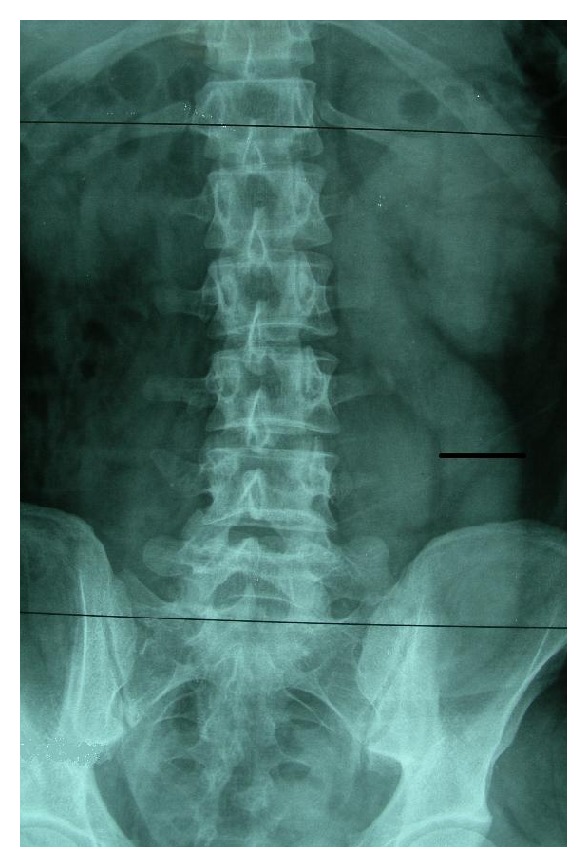
Postoperative intravenous pyelography.

IVP revealed residual dilatation on the left side (Figure 5). In USG there was moderate ectasia on the left side and in scintigraphy the left kidney's contribution to filtration was 31%. Control cystoscopy was unremarkable except for the left upper lateral wall where a 2 cm ureteral nipple and a 21 French endoscope could be fitted easily into the ureter.

## 3. Discussion

In adults primary megaureter rarely causes symptoms and does not require surgery. Symptomatic cases generally admit urinary tract infection (UTI), hematuria, and flank pain. The most frequent reason for megaureter is the occurrence of an adynamic segment in the distal ureter [6]. Generally, it presents symptoms in the third or fourth decades of life. It is also usually unilateral; however, in 15–25% of cases it can be bilateral. Because the distal ureter is adynamic, it is obstructed functionally. Normal upper ureter dilatation occurs secondary to functional obstruction. In advanced cases hydroureteronephrosis occurs. IVP, renography with intravenous diuretics, and antegrade pyelography are the diagnosis methods that can be used. The adynamic ureter segment is resected and ureterocystostomy is performed by using different surgical methods as treatment. Nevertheless, the results may be far from ideal [3, 4]. Unilateral disease is more common and is frequently on the left side [7]. Ureter calculi may cause many symptoms or may be symptomless. Giant ureter stones may occur alone with the effect of urinary tract malformations or the contribution of metabolic factors. Generally open surgery is the treatment of choice for large ureter stones despite extracorporeal lithotripsy and endourologic techniques [3, 4]. The stone in this case seems to have occurred due to obstructive megaureter. In adults giant ureter stones may be related to tuberculosis [8], ureterocele [9], or prolapsed benign ureter polyp [10].

A few incidences of calculus in megaureter cases have been reported in the literature [11]. However in that study nephroureterectomy was performed because of total loss of kidney function. In our case kidney function was good in spite of the ureter stone and the obstructed megaureter was treated. Ureterolithotomy and direct nipple ureteroneocystostomy were performed. Although left renal function in DMSA scintigraphy in the postoperative third month was only reduced from preoperative 39.6% to postoperative 31%, the treatment was considered as effective because there was no proliferation in urine the culture, no reflux flow in cystogram, no stricture in endoscopy and a reduction of ureteral caliber in IVP and the appearance of the left kidney and ureter was good compared to the preoperative IVP view.

Consequently the direct nipple ureteroneocystostomy technique may be a viable treatment method for POM because of its easy appliance; there is no need to prepare a submucosal tunnel for plication and there is a high success rate.
